# Dysfonction érectile chez les patients diabétiques type 2, prévalence et gravité au Service d´Urologie du Centre Hospitalier Universitaire de Fès, Maroc: à propos de 96 cas (étude transversale)

**DOI:** 10.11604/pamj.2020.37.205.21774

**Published:** 2020-10-30

**Authors:** Mustapha Ahsaini, Jean Paul Omana, Soufiane Mellas, Jalal Eddine El Ammari, Mohammed Fadl Tazi, Mohammed Jamal El Fassi, Moulay Hassan Farih

**Affiliations:** 1 Service d´Urologie, CHU Hassan II, Faculté de Médecine et de Pharmacie (USMBA), Fès, Maroc

**Keywords:** Diabète type 2, dysfonction érectile, prévalence, complications macroangiopathiques, complications microangiopathiques, Type 2 diabetes, erectile dysfunction, prevalence, macroangiopathic complications, microangiopathic complications

## Abstract

**Introduction:**

le diabète de type 2 est une pathologie très fréquente, pouvant occasionner chez certains patients des troubles érectiles. L´objectif de cette étude est de déterminer la prévalence et la gravité de la dysfonction érectile chez le patient diabétique de type 2 consultant en urologie.

**Méthodes:**

il s'agit d'une étude transversale et descriptive, menée au service d´urologie du centre hospitalier universitaire Hassan II de Fès utilisant un auto-questionnaire englobant un test « International Index of Erectile Function-5 » reposant sur 5 questions avec des résultats biologiques sanguins de routine du patient diabétique.

**Résultats:**

au total 96 questionnaires anonymes ont été distribués, sur 12 mois de recueil. L´âge moyen était de 53,5 ans. Nous notons 35% (n=34) de fumeurs actifs. La durée d´évolution du diabète était supérieure à 10 ans pour 54% (n=52) des cas. Trois des patients traités par régime alimentaire seul, 32% (n=31) par les antidiabétiques oraux, 31% (n=30) par insulinothérapie seule et 31% (n=30) par insuline et les antidiabétiques oraux. Seul 11% (n=11) des patients avaient une HbA1C inférieure à 7%. Les patients qui déclaraient présenter des troubles érectiles représentaient 70% (n=67) des diabétiques interrogés. La proportion de patients présentant un trouble de l´érection selon « International Index of Erectile Function-5 » était de 88% (n=84) dans notre étude. L'installation des troubles érectiles avait commencé depuis plus de 3 ans pour 63% (n=60) des patients. Le début des troubles était progressif chez 90% (n=86) des patients. Le taux de dépistage était de 37.5% (n=36) dans l´étude, seulement 30% (n=29) des patients ont bénéficié des inhibiteurs de Phosphodiestérase de type 5, puis d'injection intra-caverneuse ou les deux. Chez 42% (n=40) des patients présentent soit des complications macro ou micro-angiopathiques.

**Conclusion:**

la dysfonction érectile est une affection fréquente chez les hommes diabétiques mais peu évoquée. D´où l´importance d´un dépistage systématique chez toute personne diabétique et prise en charge adéquate sur le plan sexuel mais aussi cardio-vasculaire.

## Abstract (en)

**Introduction:**

type 2 diabetes is a very common condition which, in some patients, may cause erectile dysfunction. The purpose of this study is to determine the prevalence and severity of erectile dysfunction in patients with type 2 diabetes attending the Department of Urology.

**Methods:**

we conducted a cross-sectional and descriptive study in the Department of Urology at the University Hospital Center Hassan II in Fez using a self-administered questionnaire encompassing an “International Index of Erectile Function-5” test based on 5 questions with routine blood test results in diabetic patients.

**Results:**

a total of 96 anonymous questionnaires were distributed over a 12-month period. The average age of patients was 53.5 years, 35% (n=34) of them were active smokers. Diabetes had progressed for over 10 years in 54% (n=52) of cases. Three patients were treated with diet alone, 32% (n=31) with oral antidiabetic drugs, 31% (n=30) with insulin therapy alone and 31% (n=30) with insulin and oral antidiabetic drugs. Only 11% (n=11) of patients had HbA1c below 7%. Patients reporting erectile dysfunction accounted for 70% (n=67) of surveyed patients. The proportion of patients with erectile dysfunction according to the International Index of erectile function-5 was 88% (n=84). The onset of erectile dysfunction had started more than 3 years earlier in 63% (n=60) of patients. Disorders started progressively in 90% (n=86) of patients. Screening rate was 37.5% (n=36), only 30% (n=29) of patients received phosphodiesterase type 5 inhibitors, then intracavernous injection or both. Forty-two per cent (n=40) of patients had either macro or micro-angiopathic complications.

**Conclusion:**

erectile dysfunction is a common but little suspected condition in diabetic men. Hence, the importance of systematic screening in any diabetic patient and of adequate treatment for sexual and cardiovascular disorders.

## Introduction

La dysfonction érectile (DE) est l´incapacité persistante ou répétée d´obtenir et/ou de maintenir une érection suffisante pour permettre une activité sexuelle satisfaisante évoluant depuis plus de 3 mois. Les complications imputables au diabète notamment l´hyperglycémie chronique est à l´origine de modifications endothéliales, dont la fonction principale dans le mécanisme de l´érection semble capitale par la libération de neuromédiateur chimiques responsable de relâchement des fibres musculaires lisses [1]. Le diabète est l´une de pathologie très fréquente au Maroc et en grande partie le suivi et la prise en charge sont réalisés par les médecins généralistes. A ce titre que le diabète pose un problème de santé publique avec une prévalence estimée à 5,66% soit 2 millions des diabétiques sur 34,74 millions d´habitants [2]. Le but de cette étude est de déterminer la prévalence et la gravité de la DE chez les patients diabétiques non insulino dépendants. Aux meilleurs de nos connaissances, c´est la seule étude en son genre qu´on dispose à l´échelle nationale marocaine.

## Méthodes

**Conception et cadre de l´étude:** il s´agit d´une étude transversale réalisée en 2016 auprès des patients diabétiques consultants au sein du service d´urologie du Centre Hospitalier Hassan II de Fès - Maroc, en vue de déterminer la prévalence de DE selon le test semi quantitative « International Index of Erectile Function » (IIEF-5) [3] reposant sur 5 questions précises sur le dépistage et la qualité d'érection permettant ainsi d'établir une « prévalence diagnostiquée ».

**Population d'étude:** la taille d´échantillon a été calculée selon la méthode standard en se référant sur la prévalence de dysfonction érectile dans la région de Marrakech qui était de 32,9% [4] avec 5% comme erreur standard. Au total 120 patients diabétiques non insulinodépendants consultant au service d´urologie ont participé à l´étude. Les critères d´inclusions étaient des patients diabétiques non insulinodépendants de sexe masculin, âgés entre 30 et 80 ans. Les critères d´exclusions étaient des patients diabétiques insulinodépendants, âge plus de 80 ans et moins de 30 ans.

**Collecte des données:** le recueil des données a été réalisé par un praticien avec respect d´anonymat et après avoir expliqué au patient l´objectif et l´intérêt du travail, via un questionnaire développé de la littérature, comportant des questions d´ordre sociodémographique, histoire Clinique, traitement et prise en charge du diabète ainsi que les habitudes toxiques des patients interrogés. L´évaluation des différents aspects de la sexualité masculine (érection, satisfaction, orgasme et désir) au cours des six derniers mois a été évaluée par un questionnaire semi quantitative IIEF-5. Constituée de 5 questions dont la réponse est cotée de 0 à 4 ou 5 par question [3]. Validé par des spécialistes en épidémiologie, diabétologie et d´urologie.

**Analyses statistiques:** les données recueillies ont été consignées sur une fiche d´exploitation et ont fait l´objet d´une saisie informatique, traitement des données et d´une analyse statistique en utilisant le logiciel « SPSS ». L´analyse descriptive des données était réalisées en utilisant le test de Student et test de Khi-2 selon la nature quantitative ou qualitative des données. Avec un seuil de signification de 0,05.

**Considération éthique:** le questionnaire était rempli de façon anonyme après obtention du consentement verbal du patient, on lui expliquant les objectifs et l´intérêt du travail à fin de protéger sa confidentialité. Nous tenons à informer le rédacteur en chef que ce type d´article n´est pas soumis à l´approbation de nôtre comité éthique. Toutes les règles d´éthique et d´anonymat sont respectées lors de la rédaction de cet article.

## Résultats

Dans une période de 12 mois de recueil, 120 questionnaires ont été distribué pour un échantillon de 120 patients retenus pour l´étude. Dix patients ont refusé de participer à l'étude, 14 patients n'ont pas réussi à remplir le questionnaire dans son intégralité malgré l'aide de l'investigateur (raisons personnelles, non compréhension des questions). Au total 96 questionnaires ont été analysés.

**Figure 1:**
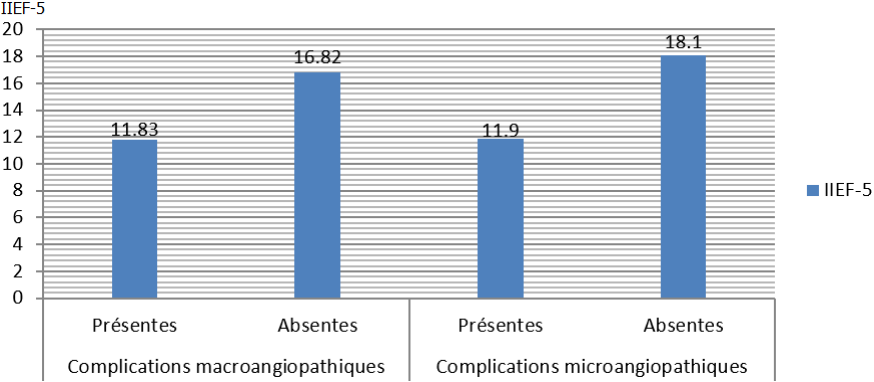
corrélation entre les complications vasculaires et le score IIEF-5

**Caractéristiques générales:** l´âge des patients interrogés était de 31 à 74 ans, avec une moyenne de 53,5 ans. Les deux tiers de nos malades n´exercent pas une activité soit en retraite ou en chômage et 76% étaient mariés. Nous avons observé que la DE était liée à l´hypertension artérielle dans 37,9% (n=36), le tabagisme dans à peu près 50% (n=48) (Tableau 1). Pour 63,5% (n=61) des patients il s´agissait d´une première consultation chez l´urologue avec comme principale plainte des troubles urinaires de bas appareil (TUBA) dans 34,4% (33), et une dysfonction érectile n´est notée que chez 11,8% d´entre eux (n=11). A noter que le délai entre les premiers signes de DE et la première consultation chez un urologue-andrologue est plus d´un an pour près des deux tiers de l´échantillon étudié. Nous avions constaté que 40% (n=38) des personnes interrogées présentent déjà des complications microangiopathiques (Tableau 2), et que parmi les patients ayant des complications microangiopathiques, l´IIEF-5 moyen était de 11,9 (c´est-à-dire une DE modéré) contre un score moyen à 18,1 (c´est-à-dire une DE légère) chez les patients n´ayant pas d´atteinte microangio pathique connue (Figure 1).

**Tableau 1:** facteurs de risque liés à la dysfonction érectile

Antécédents médicaux et chirurgicaux	La prévalence en moyenne(%)
**Hypertension artérielle**	37,9% (n=36)
**Dyslipidémie**	34,7% (n=62)
**Pathologie prostatique**	75,4% (n=72)
**Chirurgie urologique**	15,4% (n=15)
**Tabagisme**	50% (n=48)
**Alcoolisme**	6,3% (n=6)
**Surpoids**	77% (74)

**Tableau 2:** distribution des complications vasculaires de la population étudiée

		Complications microangiopathiques		
		NON	OUI	TOTAUX
**Complications macroangiopathiques**	NON	**56 (58%)**	17 (18%)	73 (76%)
	OUI	2 (2%)	21 (22%)	23 (24%)
**TOTAUX**		58 (60%)	**38 (40%)**	100%)

**La prévalence de la DE:** dans la population globale des patients qui ont participé à l´étude, 67,7% (n=65) déclaraient présenter des troubles érectiles. Pourtant, la proportion de patients présentant un trouble érectile selon l'IIEF-5 était de 88,54% (n= 85). C´est à dire que 23,52% (n=20) présentaient des troubles érectiles mais ne déclaraient pas, dans ce sous-groupe les raisons de non déclarations soit: problème non essentiel 41%, honte 35%, ou bien semble normal vue l´âge. Le plus souvent il s´agit des troubles érectiles légères à modérés 38,54% (n=37) (Tableau 3). A noter également qu´un taux de dépistage de DE par le médecin traitant avant nôtre étude était faible de 37,5% (n=36).

**Tableau 3:** déclaration et prévalence de la DE selon la sévérité

IIEF-5 et sévérité	Déclaration TE	Prévalence IIEF-5	Pas de déclaration de TE	% prévalence (N=96)
IIEF5 < 8 : **DE sévère**	22	22	0	22,92%
8 < IIEF5 < 11: **DE modérée**	20	20	0	20,83%
12 < IIEF5 < 16: **DE légère à modérée**	21	37	16	38,54%
17 < IIEF5 < 21: **DE légère**	2	6	4	6,25%
22 < IIEF5 < 25: **DE -**	2	11	-	11,46%
**TOTAUX, dont: DE +** (IIEF-5)	**67** 65	**96** 85	**20 20**	**100% 88,54%**

TE : troubles érectiles. DE- : pas de dysfonction érectile. DE+ : dysfonction érectile présente

**La prise en charge de la DE:** suite à cette découverte des troubles érectiles, seulement le tiers des médecins traitants sollicitaient une consultation spécialisée d´un urologue, et encore rares sont ceux qui sollicitaient un avis de cardiologie. Mais seulement 30% (n=25) des patients ont bénéficié d´un traitement à base des inhibiteurs de phospho-di-estérase type 5, injection intra-caverneuse chez 2 patients, les deux modalités de traitements chez un seul patient.

## Discussion

La dysfonction érectile est un trouble de la sexualité fréquent dans le monde, mais reste insuffisamment explorée. Le diabète en particulier du type 2 est l´une de pathologie redoutable par son hyperglycémie chronique, qui peut occasionner une dysfonction érectile par l´altération de l´endothélium vasculaire. Le diabète type 2 ou non insulinodépendant qui attire notre attention, représente environ 90% des cas, il survient le plus souvent autour de la cinquantaine chez des sujets le plus souvent en surpoids. Le choix des limites d'âge d'inclusion introduit également un biais de sélection mais qui était justifié: la limite inférieure permet d´exclure au maximum les diabétiques de type 1, tandis que la limite supérieure élimine les taux de prévalence élevés de trouble érectile connus chez le sujet âgé indépendamment du diabète [1]. Le rôle du diabète comme facteur de risque de trouble érectile a été prouvé par de larges études à travers le monde. La prévalence des troubles érectiles chez le diabétique de type 2 est élevée d'après les enquêtes épidémiologiques récentes, et cela montre qu'un déséquilibre du diabète majore le risque de trouble érectile. Quelques études ont récemment rapporté des taux de prévalence de la dysfonction érectile dans la population générale variables selon les pays: en France, environ 1 homme sur 3 (31,6%) [5] présenterait une dysfonction érectile; en Turquie, 69,2% [6] chez les plus de 40 ans; en Allemagne, 19,2% [7] chez les hommes âgés de 30 à 70 ans. Nous avons constaté que 88,54% (n=85) des patients étaient atteints de dysfonction érectile, alors que la série de Lokrou et Soumahoro [8] réalisée en Côte d´Ivoire en 2011 retrouvait une prévalence de 86,1%. Dans la série tunisienne de Bayar *et al*. [9], la prévalence était de 83,5%. Tandis que dans la série française de Leplae [10] publiée en 2014 et utilisant le questionnaire IIEF-5, elle retrouvait une prévalence de 72%. Une étude israélienne de Roth *et al*. [11] réalisée en 2003 a démontré une prévalence de 76,5%. Les chiffres de prévalence retrouvés dans cette étude sont donc assez proches de ceux de nos voisins africains, et globalement supérieurs à ceux retrouvés dans la population européenne. La notion d´âge à prendre avec beaucoup de considération, c´est pourquoi l´âge de patients qui ont participé à cette étude est entre 50 et 59 ans avec un âge moyen de 53,5 ans±11,5. L´âge moyen de survenue de la dysfonction érectile chez le diabétique de type 2 au Burkina Faso était de 52,3 ans [12], et 58,8 ans chez les tunisiens [9], 57 ans chez les guinéens [13] et 52,8 ans chez les ivoiriens [8]. L´influence de l´âge sur la prévalence de la dysfonction érectile est bien établie avec une augmentation de la sévérité de la dysfonction érectile était classiquement retrouvée dans la littérature [5]. Le prostatisme était le principal motif de consultation 34,4% (n=33) de cas. Une dysfonction érectile était citée comme motif de consultation par 11,8% (n=11) des patients. Ces résultats concordent avec ceux rapportés par l´étude française de Droupy *et al*. [14]: un problème de prostate comme principal motif de consultation indiqué par les patients (62,2%), unique motif dans 45,4% des cas. Le trouble sexuel venait en deuxième position, avec 14,2% des patients. La dysfonction érectile fût découverte chez la majorité de ces patients grâce à l´interrogatoire sur le maintien de la vie sexuelle en général et l´IIEF-5.

La mise en évidence d´une dysfonction érectile et son intensité (légère, modérée ou sévère) reposait sur l´IIEF-5 [3]. Ainsi, par cet outil, nous trouvions que la forme modérée (8 < IIEF < 16) était la plus fréquente, représentant 59.37% (n=57) des patients. La série guinéenne de N.M. Baldé objectivait par contre une plus large proportion des formes sévères avec 54,45% [13], en utilisant toujours le même questionnaire de l´IIEF-5. Tandis qu´en France, dans la série de Droupy *et al*. la forme légère est la plus représentée [14]. Il a été susmentionné les fréquences élevées de dysfonction érectile chez des patients diabétiques, hypertendus, tabagiques, alcooliques et ayant des antécédents de chirurgie pelvienne, périnéale ou pénienne. Ce qui nous permet de dire sans pouvoir le démontrer que ces différents éléments constitueraient des facteurs de risque de dysfonction érectile à la suite à des neuropathies et angiopathies périphériques. L´étude de Bacon *et al*. [15], dans une large enquête prospective, avait permis d´observer des risques relatifs de développer une dysfonction érectile respectivement de 1,9 et 1,5 en cas d´obésité et de tabagisme. Parmi les patients souffrant de dysfonction érectile, 62,4% (n=65), avaient parlé de leur trouble à un médecin. La série française de Droupy *et al*. [14] rapportent 58.8% de patients ayant déclaré leur dysfonction érectile au médecin. Dans le groupe ayant fait part de leur trouble au médecin traitant, le taux de traitement était de 100% (n=28), alors qu´il était de 30% (n=29) sur l´ensemble des patients. Droupy *et al*. [14] ont trouvé des résultats différents, un taux de traitement plus bas 45,1% parmi ceux ayant fait part de leur trouble au médecin et un taux de 25% sur l´ensemble des patients. La durée de l´évolution du diabète était supérieure à 10 ans pour 54% (n=52) de cas, alors que dans la série Ivoirienne de Lokrou et Soumahoro [8], une évolution de plus de 10 ans est retrouvée chez 75,9% des patients diabétiques présentant une dysfonction érectile. L´étude française de Leplae retrouvait une moyenne d´évolution de 8 ans [10]. La durée d´évolution du diabète est liée à une plus forte prévalence de la maladie [11]. Concernant l´équilibre du diabète, on retrouvait dans notre étude un taux moyen d´HbA1c de 9,1%. Ces chiffres demeurent assez proches de ceux repris dans l´étude sénégalaise de Gueye *et al*. qui rapportait un taux moyen de 8.7% [16]. Par ailleurs, la série française de Leplae [10] retrouvait une moyenne nettement plus basse avec un taux de 7,73%.

De ce fait, nous avons pu remarquer qu'à l'instar d'autres études, un taux d'HbA1C élevé serait lié à une dysfonction érectile plus sévère. Comme toute pathologie, il a été noté quelques complications du genre macroangiopathiques dans 24% des patients, microangiopathiques dans 40% et des complications mixtes dans 22% de cas. Dans la série américaine de Reriani *et al*. réalisée en 2016, les complications macroangiopathiques étaient retrouvées chez 60% des patients étudiés tandis que les complications microangiopathiques étaient de 52% [17]. L'étude de notre population montre un taux de dépistage de DE par le médecin traitant faible de 37,5% (n=36), et qui rejoint celle retrouvée en France en 2004, où on retrouve dans une population constituée uniquement de diabétiques un taux de dépistage de 30% (Giuliano *et al*. 2004) [18]. Ainsi, on comprend que les recommandations pour la prise en charge du diabète mentionnent la nécessité du dépistage des troubles érectiles par le médecin généraliste. La dysfonction érectile pourra même être une symptomatologie d´une maladie cardiovasculaire, voilà pourquoi dans l´actualisation du IIIe consensus de Princeton en 2005, les auteurs [19] ont noté que « tout homme présentant une dysfonction érectile sans cause évidente devrait être dépisté pour les maladies cardiovasculaires ». Le point faible de notre étude c´est le nombre réduit de notre échantillon, avec un biais de sélection dû à la consultation spécialisé en urologie de ces patients diabétique.

## Conclusion

Notre étude a mis en évidence une carence manifeste dans le dépistage et la prise en charge de cette complication liée au diabète malgré des connaissances scientifiques solides. Outre la prévalence élevée des troubles érectiles, cette étude montre également que les diabétiques attendent que leurs médecins engagent en premier la discussion sur ces troubles sexuels, car la sexualité reste encore jusqu´à preuve du contraire un tabou dans le contexte arabo-musulman. Ainsi, la dysfonction érectile peut représenter un signal d´alarme précoce de maladie cardiovasculaire, et représente aussi une opportunité pour les cliniciens à fin d´améliorer la santé des hommes avant leur première manifestation cardiovasculaire. Une meilleure prise en charge, une meilleure information et une meilleure orientation permettraient à ces patients porteurs d'une maladie chronique, de réduire le risque de rupture thérapeutique, de comprendre le lien qu'il existe entre diabète et trouble érectile.

### Etat des connaissances sur le sujet

- La prévalence des troubles érectiles chez le diabétique de type 2 est élevée d'après les enquêtes épidémiologiques récentes;
- La dysfonction érectile peut représenter un signal d´alarme précoce de maladie cardiovasculaire, et représente aussi une opportunité pour les cliniciens à fin d´améliorer la santé des hommes avant leur première manifestation cardiovasculaire.

### Contribution de notre étude à la connaissance

- Aux meilleurs de nos connaissances, c´est la seule étude en son genre qu´on dispose à l´échelle nationale marocaine sur la prévalence et la gravité de la DE chez les patients diabétiques non insulino dépendants;
- Cette étude montre également que les diabétiques attendent que leurs médecins engagent en premier la discussion sur ces troubles sexuels, car la sexualité reste encore jusqu´à preuve du contraire un tabou le contexte arabo-musulman.

## Remerciements

Remerciement spécial à Mr Abdelilah Benslimane du laboratoire d´épidémiologie et recherche clinique de la faculté de médecine et de pharmacie de Fès pour son assistance et son aide précieuse pour l´analyse et l´exploitation des données.
